# Supplementary material for: Individual variation of the masticatory system dominates 3D skull shape in the herbivory-adapted marsupial wombats
Source: Front Zool. 2019 Nov 1;16:41. doi: 10.1186/s12983-019-0338-5 (PMC6824091; doi:10.1186/s12983-019-0338-5)
Supplement: Supplementary file 1 — Additional file 1. Additional analyses: 1A) Procrustes ANOVA tables for sexual dimorphism of Shape~Sex and Size~Sex; also compilable from the 06-Standard Analysis script on GitHub; 1B) Comparison of all analyses in Table 1 of the main manuscript with the full landmark dataset (a total of 761 and 577 for cranium and mandible, respectively) compared to the dataset with fixed landmarks and curve semilandmarks (261 and 142, respectively) and the dataset with only fixed landmarks (a total of 65 and 35, respectively). There are no differences between the analyses with and without surface semilandmarks (top and bottom of the table). There are very few differences between the analyses with all landmarks vs. analyses with just fixed landmarks; these exclusively concern cases where one analysis is not significant and the other is above 0.01, and thus very close to the significance cut-off of 0.05 already. [file 12983_2019_338_MOESM1_ESM.docx]

# Additional File 1: Supplementary Analyses

**Additional File 1A:** Procrustes ANOVA for sexual dimorphism of Shape~Sex and Size~Sex; also compilable from the 06-Standard Analysis script on GitHub.

**Shape~Sex, Southern Hairy-nosed wombat,** **cranium**

|  | Df | SS | MS | Rsq | F | Z | Pr(>F) |
| --- | --- | --- | --- | --- | --- | --- | --- |
| Sex | 1 | 0.001 | 0.001 | 0.033 | 0.572 | -1.341 | 0.925 |
| Residuals | 17 | 0.038 | 0.002 | 0.967 |  |  |  |
| Total | 18 | 0.039 |  |  |  |  |  |

**Shape~Sex, Southern Hairy-nosed wombat,** **mandible**

|  | Df | SS | MS | Rsq | F | Z | Pr(>F) |
| --- | --- | --- | --- | --- | --- | --- | --- |
| Sex | 1 | 0.001 | 0.001 | 0.044 | 0.74 | -0.757 | 0.766 |
| Residuals | 16 | 0.032 | 0.002 | 0.956 |  |  |  |
| Total | 17 | 0.034 |  |  |  |  |  |

**Shape~Sex, common wombat,** **cranium**

|  | Df | SS | MS | Rsq | F | Z | Pr(>F) |
| --- | --- | --- | --- | --- | --- | --- | --- |
| Sex | 1 | 0.002 | 0.002 | 0.051 | 0.811 | -0.462 | 0.673 |
| Residuals | 15 | 0.034 | 0.002 | 0.949 |  |  |  |
| Total | 16 | 0.036 |  |  |  |  |  |

**Shape~Sex, common wombat,** **mandible**

.

|  | Df | SS | MS | Rsq | F | Z | Pr(>F) |
| --- | --- | --- | --- | --- | --- | --- | --- |
| Sex | 1 | 0.002 | 0.002 | 0.059 | 0.753 | -0.623 | 0.709 |
| Residuals | 12 | 0.027 | 0.002 | 0.941 |  |  |  |
| Total | 13 | 0.028 |  |  |  |  |  |

**Size~Sex, Southern Hairy-nosed wombat,** **cranium**

|  | Df | SS | MS | Rsq | F | Z | Pr(>F) |
| --- | --- | --- | --- | --- | --- | --- | --- |
| Sex | 1 | 2801.749 | 2801.749 | 0.039 | 0.696 | 0.36 | 0.431 |
| Residuals | 17 | 68428.967 | 4025.233 | 0.961 |  |  |  |
| Total | 18 | 71230.715 |  |  |  |  |  |

**Size~Sex, Southern Hairy-nosed wombat,** **cranium**

|  | Df | SS | MS | Rsq | F | Z | Pr(>F) |
| --- | --- | --- | --- | --- | --- | --- | --- |
| Sex | 1 | 83.841 | 83.841 | 0.002 | 0.039 | -0.981 | 0.863 |
| Residuals | 16 | 34431.753 | 2151.985 | 0.998 |  |  |  |
| Total | 17 | 34515.593 |  |  |  |  |  |

**Size~Sex, common wombat,** **cranium**

|  | Df | SS | MS | Rsq | F | Z | Pr(>F) |
| --- | --- | --- | --- | --- | --- | --- | --- |
| Sex | 1 | 232.023 | 232.023 | 0.002 | 0.025 | -1.182 | 0.875 |
| Residuals | 15 | 139007.711 | 9267.181 | 0.998 |  |  |  |
| Total | 16 | 139239.734 |  |  |  |  |  |

**Size~Sex, common wombat,** **mandible**

|  | Df | SS | MS | Rsq | F | Z | Pr(>F) |
| --- | --- | --- | --- | --- | --- | --- | --- |
| Sex | 1 | 699.007 | 699.007 | 0.015 | 0.182 | -0.244 | 0.684 |
| Residuals | 12 | 46121.790 | 3843.483 | 0.985 |  |  |  |
| Total | 13 | 46820.798 |  |  |  |  |  |

**Additional File 1B:** Results of all analyses in Table 1 of the main manuscript with the full landmark dataset (a total of 761 and 577 for cranium and mandible, respectively) compared to the dataset with fixed landmarks and curve semilandmarks (261 and 142, respectively ) and the dataset with only fixed landmarks (a total of 65 and 35, respectively). There are no differences between the analyses with and without surface semilandmarks (top and bottom of the table). There are very few differences between the analyses with all landmarks vs. analyses with just fixed landmarks; these exclusively concern cases where one analysis is not significant and the other is above 0.01, and thus very close to the significance cut-off of 0.05 already.

|  | | *n* | | PC1 % | | Allometry | | | | | | 2BPLS | | | | PLS1 scores~PC1 scores | | | | | | PLS1 scores~Csize | | | | | |  |  |
| --- | --- | --- | --- | --- | --- | --- | --- | --- | --- | --- | --- | --- | --- | --- | --- | --- | --- | --- | --- | --- | --- | --- | --- | --- | --- | --- | --- | --- | --- |
|  | |  | |  | | R^2^ | | F | | *p* | | *n* | | r-PLS | | cor | | t | | *p* | | cor | | t | | *p* | | | |
| Cranium | | | | | | | | | | | | | | | | | | | | | | | | | | | |  |  |
| Common | | 24 | | 23.49 | | 0.05 | | 1.133 | | 0.283 | | 18 | | 0.85 | | -0.83 | | -5.93 | | **0.000** | | -0.046 | | -0.19 | | 0.86 | |  |  |
| NHNW | | 23 | | 21.56 | | 0.11 | | 3.079 | | **0.001**** | | 11 | | 0.93 | | -0.7 | | -2.93 | | **0.017** | | -0.57 | | -2.1 | | 0.07 | |  |  |
| SHNW  PC2, SHNW | | 24 | | 25.92 | | 0.049 | | 1.138 | | 0.305 | | 19 | | 0.93 | | 0.19  0.89 | | 0.81  8.05 | | 0.43  **0.000** | | -0.27 | | -1.16 | | 0.26 | |  |  |
| Mandible | | | | | | | | | | | | | | | | | | | | | | | | | | | |  |  |
| Common | | 21 | | 23.79 | | 0.59 | | 1.188 | | 0.263 | |  | |  | | 0.99 | | 22.508 | | **0.000** | | 0.09 | | 0.36 | | 0.72 | |  |  |
| NHNW | | 13 | | 25.88 | | 0.118 | | 1.337 | | 0.148 | |  | |  | | -0.72 | | -3.16 | | **0.012** | | -0.58 | | -2.15 | | 0.06 | |  |  |
| SHNW | | 21 | | 18.93 | | 0.091 | | 1.905 | | **0.019** | |  | |  | | 0.89 | | 7.67 | | **0.000** | | -0.6 | | -3.08 | | **0.007** | |  |  |
| Below – fixed landmarks only | | | | | | | | | | | | | | | | | | | | | | | | | | | |  |  |
|  | | *n* | | PC1 % | | Allometry | | | | | | 2BPLS | | | | PLS1 scores~PC1 scores | | | | | | PLS1 scores~Csize | | | | | |  |  |
|  | |  | |  | | R^2^ | | F | | *p* | | *n* | | r-PLS | | cor | | t | | *p* | | cor | | t | | *p* | | | |
| Cranium | | | | | | | | | | | | | | | | | | | | | | | | | | | |  |  |
| Common | | 24 | | 18.5 | | 0.063 | | 1.48 | | 0.069 | | 18 | | 0.91 | | 0.75 | | 4.7 | | **0.000** | | -0.022 | | -0.08 | | 0.9 | |  |  |
| NHNW | | 23 | | 18 | | 0.108 | | 2.54 | | **0.001**** | | 11 | | 0.98 | | 0.22 | | 0.7 | | **0.5** | | 0.73 | | 3.2 | | **0.01** | |  |  |
| SHNW  PC2, SHNW | | 24 | | 28.2 | | 0.059 | | 1.38 | | 0.163 | | 19 | | 0.94 | | 0.52  0.77 | | 2.52  5.0 | | **0.022**  **0.000** | | -0.04 | | -.014 | | 0.88 | |  |  |
| Mandible | | | | | | | | | | | | | | | | | | | | | | | | | | | |  |  |
| Common | | 21 | | 19.7 | | 0.77 | | 1.58 | | 0.057 | |  | |  | | 0.9 | | -8.1 | | **0.000** | | -0.06 | | -0.23 | | 0.8 | |  |  |
| NHNW | | 13 | | 20.5 | | 0.11 | | 1.292 | | 0.158 | |  | |  | | 0.26 | | 0.80 | | 0.44 | | 0.77 | | 3.69 | | **0.004** | |  |  |
| SHNW | | 21 | | 19 | | 0.075 | | 1.533 | | 0.085 | |  | |  | | 0.63 | | 3.35 | | **0.004** | | -0.40 | | -1.87 | | 0.08 | |  |  |
| Below – fixed and curve landmarks | | | | | | | | | | | | | | | | | | | | | | | | | | | |  |  |
|  | | *n* | | PC1 % | | Allometry | | | | | | 2BPLS | | | | PLS1 scores~PC1 scores | | | | | | PLS1 scores~Csize | | | | | |  |  |
|  | |  | |  | | R^2^ | | F | | *p* | | *n* | | r-PLS | | cor | | t | | *p* | | cor | | t | | *p* | | | |
| Cranium | | | | | | | | | | | | | | | | | | | | | | | | | | | |  |  |
| Common | | 24 | | 24.4 | | 0.055 | | 1.284 | | 0.171 | | 18 | | 0.84 | | -0.89 | | -7.72 | | **0.000** | | -0.11 | | -0.46 | | 0.65 | |  |  |
| NHNW | | 23 | | 22.5 | | 0.111 | | 2.6 | | **0.004** | | 11 | | 0.92 | | -0.73 | | -3.19 | | **0.01** | | -0.53 | | -1.87 | | 0.09 | |  |  |
| SHNW  PC2, SHNW | | 24 | | 26.1 | | 0.068 | | 1.6 | | 0.107 | | 19 | | 0.93 | | 0.36 | | 1.6 | | 0.128 | | -0.26 | | -1.1 | | 0.29 | |  |  |
| Mandible | | | | | | | | | | | | | | | | | | | | | | | | | | | |  |  |
| Common | | 21 | | 22.7 | | 0.062 | | 1.258 | | 0.224 | |  | |  | | 0.95 | | 13.21 | | **0.000** | | 0.033 | | 0.13 | | 0.898 | |  |  |
| NHNW | | 13 | | 21.3 | | 0.123 | | 1.397 | | 0.116 | |  | |  | | -0.72 | | -3.07 | | **0.013** | | -0.56 | | -2.03 | | 0.07 | |  |  |
| SHNW | | 21 | | 18.4 | | 0.081 | | 1.668 | | **0.024** | |  | |  | | 0.84 | | 6.41 | | **0.000** | | -0.53 | | -2.55 | | **0.021** | |  |  |
