# Supplementary figures and images for: Individual variation of the masticatory system dominates 3D skull shape in the herbivory-adapted marsupial wombats
Source: Front Zool. 2019 Nov 1;16:41. doi: 10.1186/s12983-019-0338-5 (PMC6824091; doi:10.1186/s12983-019-0338-5)

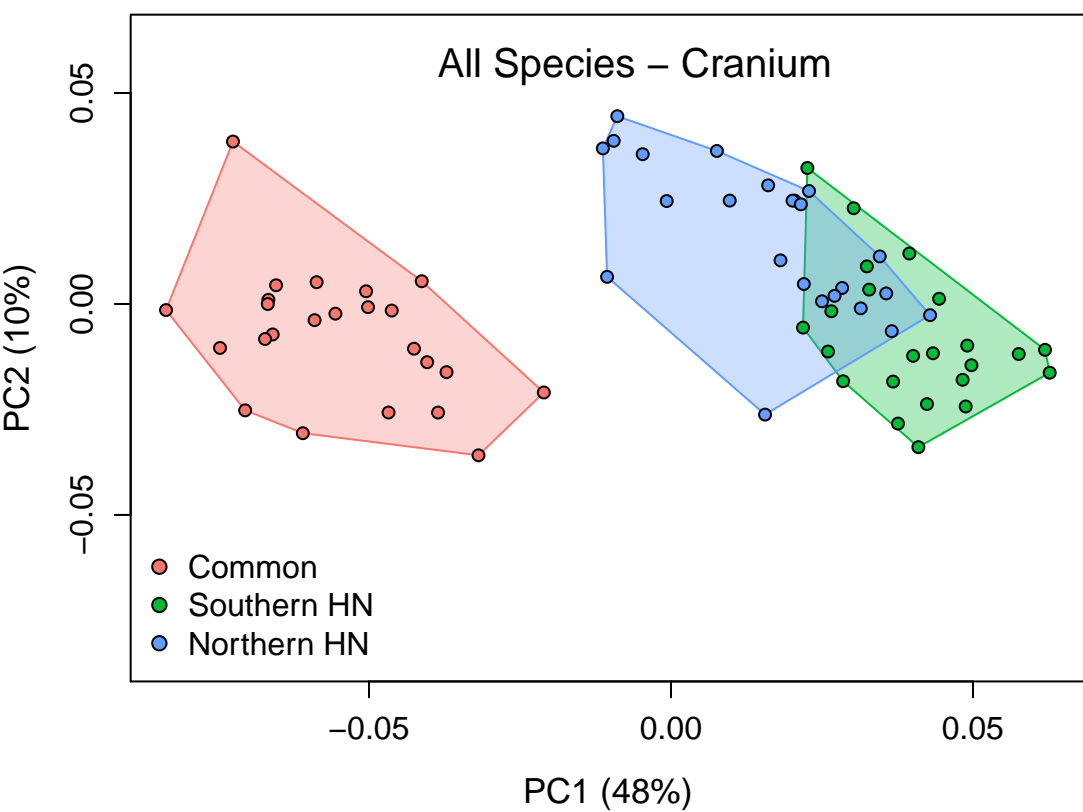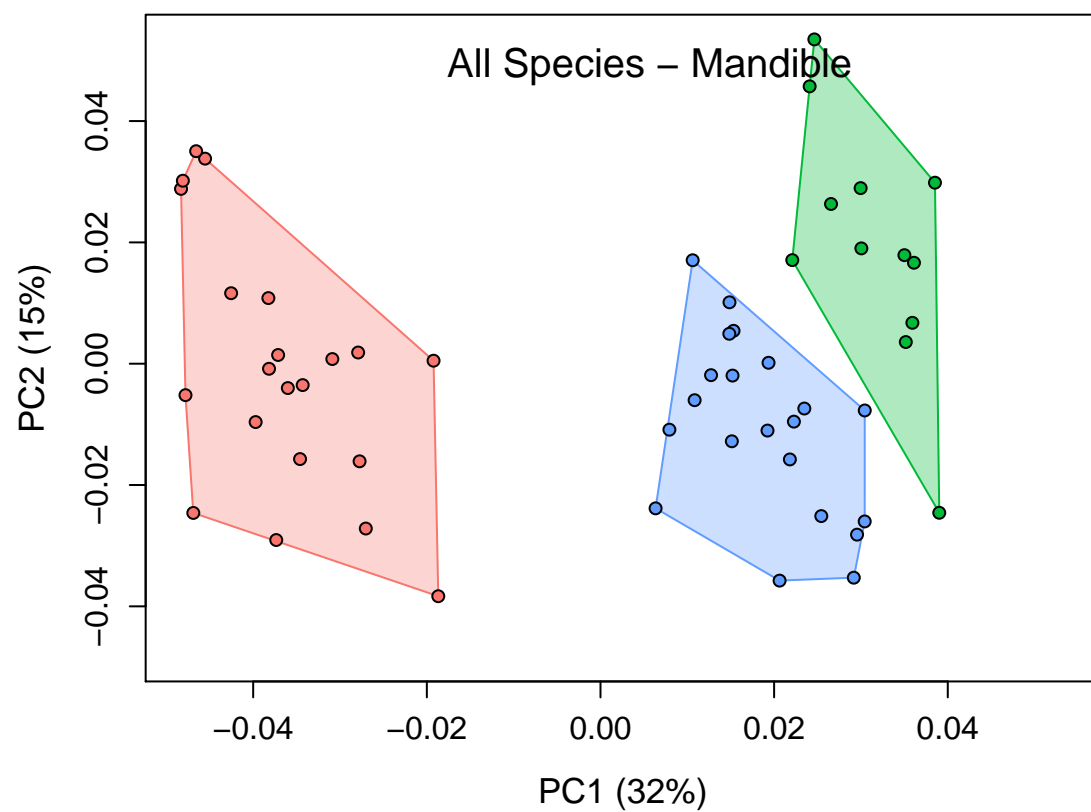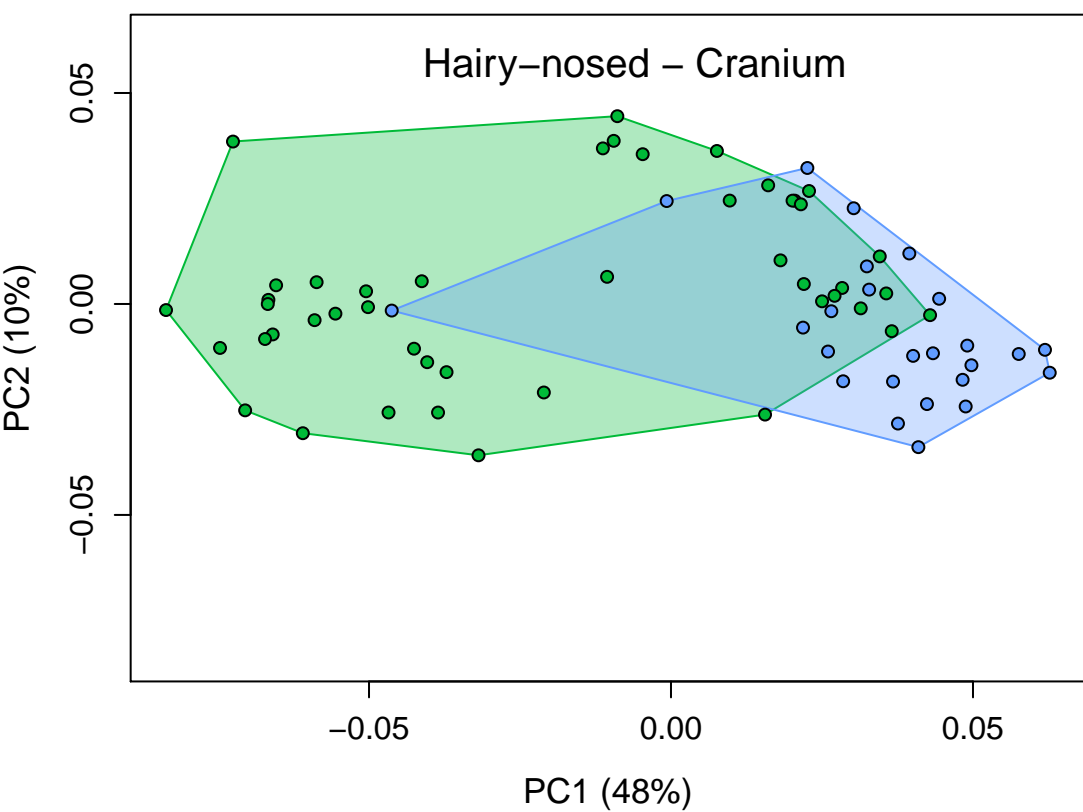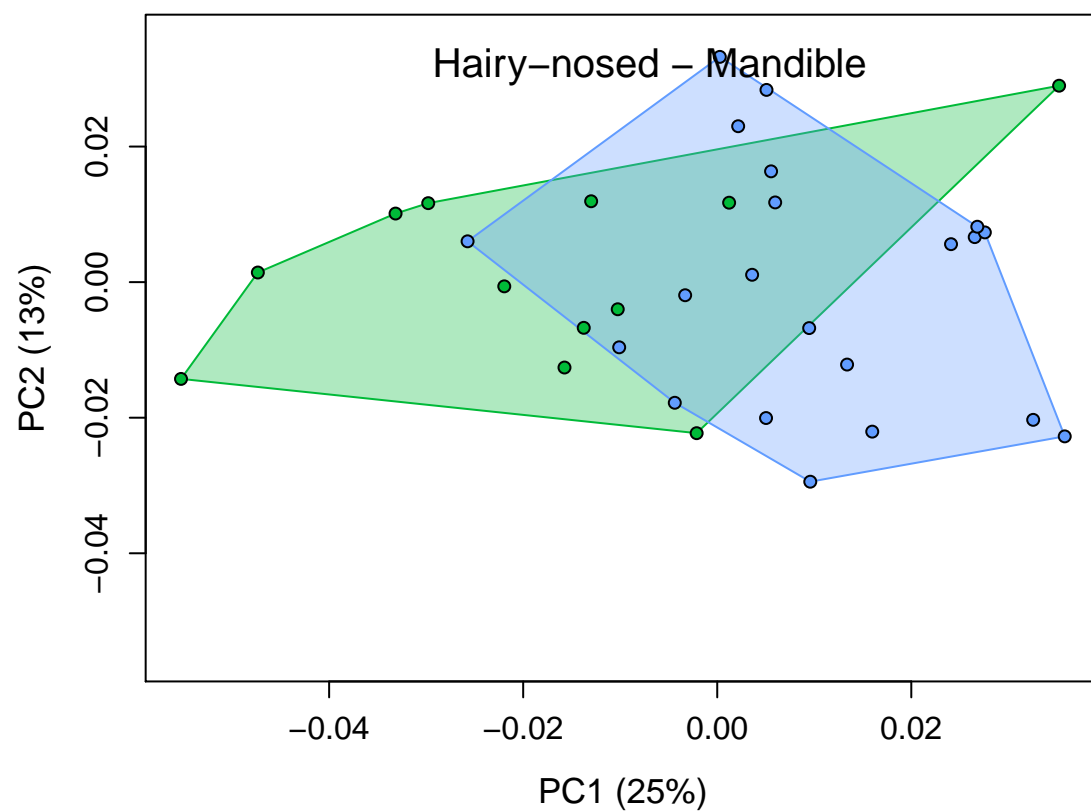

Supplement: Supplementary file 2 — Additional file 2. Principal Component 1 vs. 2 plots of cranial (left) and mandibular (right) shapes, showing distributions of specimens in the all-wombat (above) and hairy-nosed wombat (below) sample, in a PCA of residual coordinates from a regression of shape against size. [file 12983_2019_338_MOESM2_ESM.pdf]
