# Supplementary material for: Individual variation of the masticatory system dominates 3D skull shape in the herbivory-adapted marsupial wombats
Source: Front Zool. 2019 Nov 1;16:41. doi: 10.1186/s12983-019-0338-5 (PMC6824091; doi:10.1186/s12983-019-0338-5)
Supplement: Supplementary file 3 — Additional file 3. Visual representations of shape variation in the specimen pairs tested in the landmark tests. Spheres are the position of one landmark of one shape, lines represent the displacement of the same landmark in the other shape. Colour heat reflects displacement magnitude (red/yellow = high/low displacement). The comparisons have no specified direction, so that shapes were compared so that the landmarks on the zygomatic arch (cranium)/incisor root area (mandible) were pointing outwards in all specimens for ease of comparison. A, Cranium; B, Mandible. [file 12983_2019_338_MOESM3_ESM.pdf]

Additional File 3: Representations of shape variation in the specimen pairs tested in the landmark tests. Spheres are the position of one landmark of one shape, lines represent the displacement of the same landmark in the other shape. Colour heat reflects displacement magnitude (red/yellow = high/low displacement). The comparisons have no specified direction, so that shapes were compared so that the landmarks on the zygomatic arch (cranium)/incisor root area (mandible) were pointing outwards in all specimens for ease of comparison. A, Cranium; B, Mandible

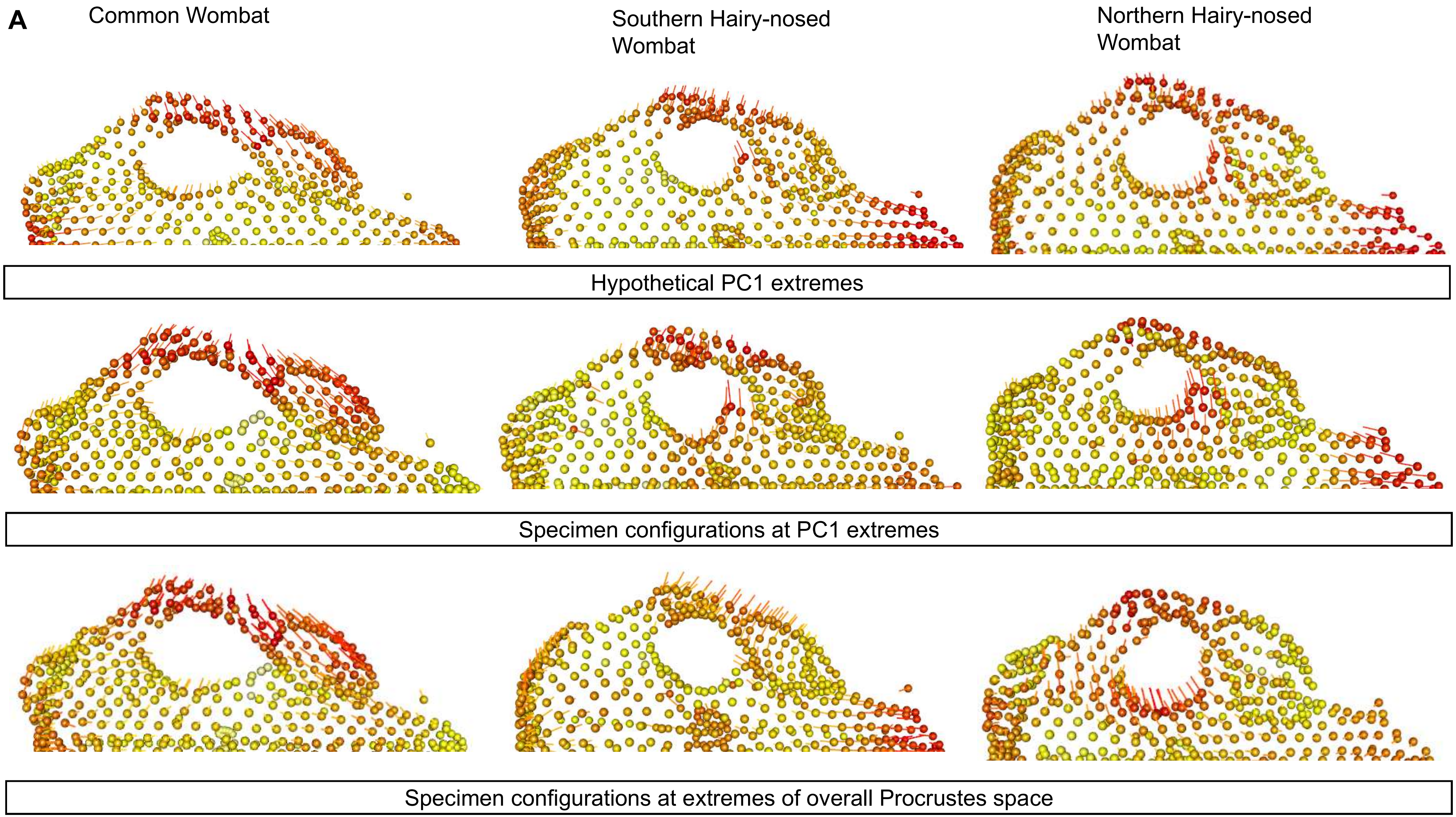

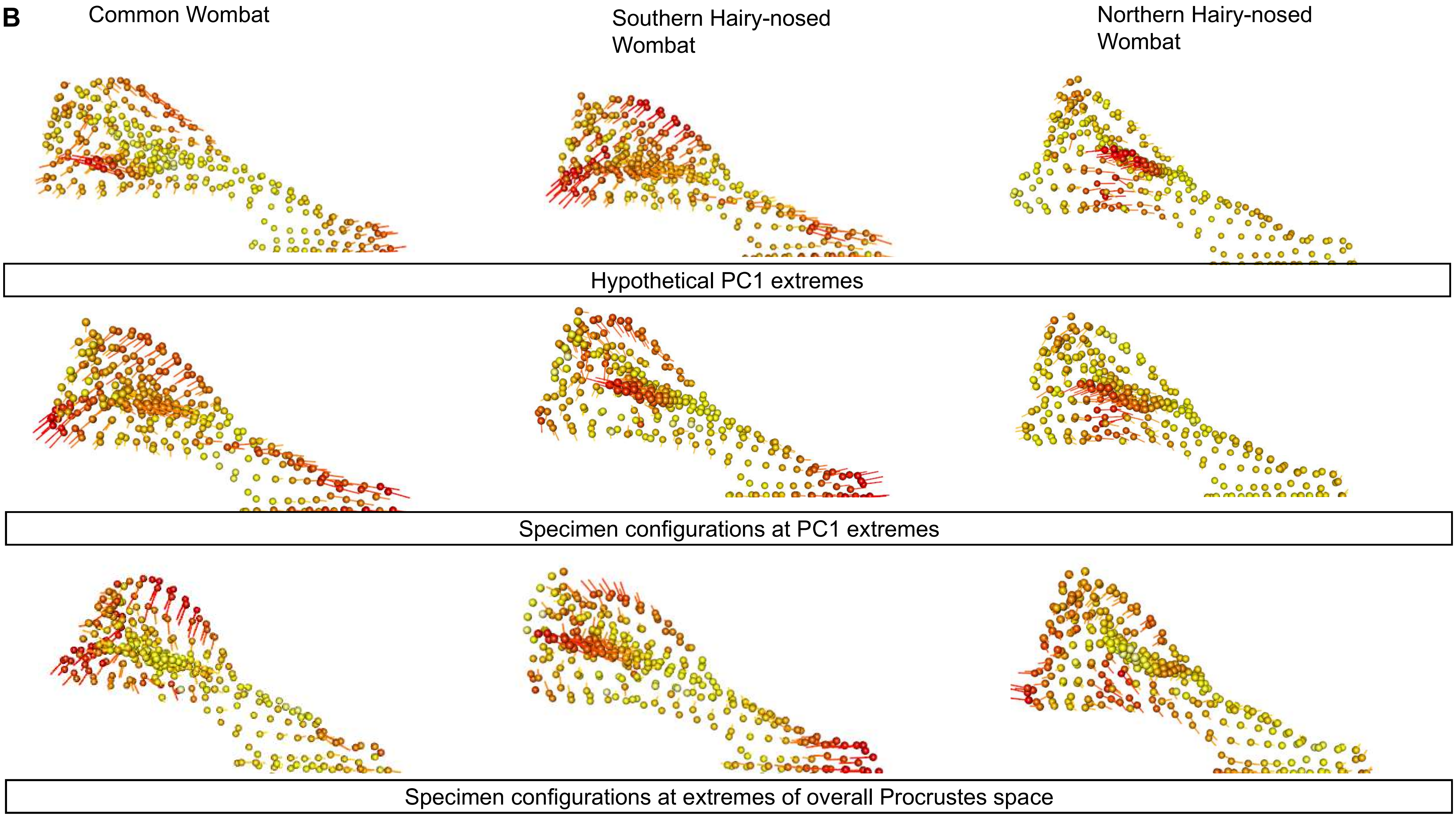
B
Common Wombat
Southern Hairy-nosed Wombat
Northern Hairy-nosed Wombat
Hypothetical PC1 extremes
Specimen configurations at PC1 extremes
Specimen configurations at extremes of overall Procrustes space
