# Supplementary material for: Individual variation of the masticatory system dominates 3D skull shape in the herbivory-adapted marsupial wombats
Source: Front Zool. 2019 Nov 1;16:41. doi: 10.1186/s12983-019-0338-5 (PMC6824091; doi:10.1186/s12983-019-0338-5)
Supplement: Supplementary file 5 — Additional file 5. Description of all landmarks used to capture the shape of the cranium and mandible. [file 12983_2019_338_MOESM5_ESM.docx]

**Additional File 7:** Description of all landmarks used to capture the shape of the cranium and mandible.

| **Type (Fixed / Curve / Patch)** | **Name** | **Side (Right / Left / Midline)** | **Location** |
| --- | --- | --- | --- |
| **Cranium** | | | |
| Fixed | Nasospinale | Mid | Medial point immediately inferior to the nasal opening |
| Fixed | Nasal bone | Mid | Medial and most anterior point on the nasal bone |
| Fixed | Nasal aperture | R / L | Most lateral point on the nasal aperture |
| Fixed | End of nasomaxillary | R / L | Point where the nasomaxillary suture ends on the rostrum |
| Fixed | Incisor | R / L | Point immediately inferior to the outer edge of the incisor |
| Fixed | Origin of incisors (inferior) | Mid | Most medial and anterior point on the ventral cranium where the incisors originate |
| Fixed | Incisive foramen | R / L | Most anterior point on the incisive foramen |
| Fixed | PM / M1 | R / L | Point between the origin of the PM and M1 on the lateral alveolar margin |
| Fixed | M1 / M2 | R / L | Point between the origin of the M1 and M2 on the lateral alveolar margin |
| Fixed | M2 / M3 | R / L | Point between the origin of the M2 and M3 on the lateral alveolar margin |
| Fixed | M3 / M4 | R / L | Point between the origin of the M3 and M4 on the lateral alveolar margin |
| Fixed | Infraorbital foramen | R / L | Point directly below the infraorbital foramen |
| Fixed | Lacrimal tubercle | R / L | Most lateral point on the lacrimal tubercle |
| Fixed | Fronto-nasal suture | Mid | Point where the internasal suture meets the fronto-nasal suture |
| Fixed | Fronto-nasal/naso-maxillary suture | R / L | Intersection of the fronto-nasal suture and the naso-maxillary suture |
| Fixed | Postorbital process | R / L | Most lateral point on the postorbital process |
| Fixed | Masseter (anterior-most) | R / L | Point where the superior rim of the masseter attachment area begins near the anterior end of the zygomatic arch |
| Fixed | Masseter (anterior-posterior) | R / L | Point where the inferior rim of the masseter attachment area begins near the anterior end of the zygomatic arch |
| Fixed | Masseter (posterior-superior) | R / L | Point where the inferior rim of the zygomatic ends on the zygomatic suture |
| Fixed | Zygomatic bone (anterior-superior) | R / L | Most superior point on the anterior end of the zygomatic arch (near where the zygomatic dip ends) |
| Fixed | Zygomatic suture origin | R / L | Superior origin of the suture on the zygomatic process |
| Fixed | Zygomatic suture (inferior) | R / L | Intersection of the zygomatic suture and ridge of the temporal fossa |
| Fixed | End of zygomatic | R / L | Point where the superior rim of the zygomatic arch ends on the posterior rim of the cranium |
| Fixed | Temporal fossa | R / L | Most posterior point on the anterior ridge of the temporal fossa |
| Fixed | Inion | Mid | Medial and most posterior point on the dorsal surface |
| Fixed | Opisthion | Mid | Point superior to foramen magnum |
| Fixed | Basion | Mid | Point inferior to foramen magnum |
| Fixed | Paroccipital process | R / L | Most inferior point on the paroccipital process |
| Fixed | Mastoid process | R / L | Most inferior point on the mastoid process |
| Fixed | Foramen ovale | R / L | Point superior to the foramen ovale |
| Fixed | Foramen alar | R / L | Point superior to the foramen alar |
| Fixed | Pterygoid hamulus | R / L | Point immediately posterior to the pterygoid hamulus |
| Fixed | Base of vomer | Mid | Point where the vomer intersects with the inferior surface of the cranium |
| Fixed | Vomer | Mid | Medial point on the vomer (bridge joining the two sides of the basicranium) |
| Fixed | Palatal vacuity | R / L | Most anterior point on the palatal vacuity |
| Curve | Nasomaxillary suture | R / L | **[End of nasomaxillary to fronto-nasal/naso-maxillary suture]** |
| Curve | Masseter (inferior rim) | R / L | Along the inferior rim of the masseter attachment area  **[Masseter (anterior-posterior) - zygomatic suture (inferior)]** |
| Curve | Masseter (superior rim) | R / L | Along the superior rim of the masseter attachment area  **[Masseter (anterior-most) to masseter (posterior-superior)]** |
| Curve | Zygomatic dip | R / L | Along the rim of the excavated portion of the superior zygomatic arch  **[Superior zygomatic arch - lachrymal tubercle]** |
| Curve | Zygomatic suture (outer) | R / L | Along the zygomatic suture on the outer surface of the zygomatic  **[Zygomatic suture (inferior) - zygomatic suture origin]** |
| Curve | Zygomatic suture (inner) | R / L | Along the zygomatic suture on the inner surface of the zygomatic  [**Zygomatic suture origin** - **masseter (posterior-superior)**] |
| Curve | Superior rim of zygomatic arch | R / L | Along the superior rim of the zygomatic arch  [**Zygomatic suture origin** to **end of zygomatic**] |
| Curve | Postorbital process to temporal fossa | R / L | Along the boundary of the temporalis posterior [**Postorbital process** to **temporal fossa**] |
| Curve | Inion-mastoid | R / L | Along the rim of the basicranium  [**Inion** to **mastoid process**] |
| Curve | Dorsal midline 1 | Mid | [**Nasal bone** to **fronto-nasal suture**] |
| Curve | Dorsal midline 2 | Mid | [**Fronto-nasal suture** to **inion**] |
| Curve | Inion-opisthion | Mid | Along the midline of the basicranium  **[Inion – opisthion]** |
| Curve | Ventral midline 1 | Mid | [**Basion** to **base of vomer**] |
| Curve | Ventral midline 2 | Mid | [**Vomer** to **origin of incisors (inferior)**] |
| Patch | Dorsal surface | R / L | Covering the entirety of the dorsal surface, bounded by the nasomaxillary sutures, inner zygomatic sutures, and rim of the temporalis superior |
| Patch | Posterior surface | R / L | Covering the entirety of the basicranium |
| Patch | Lateral surface | R / L | Covering the lateral masseter attachment area on the zygomatic arches |
| **Mandible** | | | |
| Fixed | Infradentale | Mid | Medial and inferior point between the origin of the two incisors |
| Fixed | Incisor (superior) | R / L | Point where the incisor originates on the lateral alveolar margin |
| Fixed | Origin of incisors (superior) | Mid | Most medial and anterior point on the superior mandible where the incisors originate |
| Fixed | Mental foramen | R / L | Point just superior to the mental foramen |
| Fixed | PM / M1 | R / L | Point between the origin of the PM and M1 on the lateral alveolar margin |
| Fixed | M1 / M2 | R / L | Point between the origin of the M1 and M2 on the lateral alveolar margin |
| Fixed | M2 / M3 | R / L | Point between the origin of the M2 and M3 on the lateral alveolar margin |
| Fixed | M3 / M4 | R / L | Point between the origin of the M3 and M4 on the lateral alveolar margin |
| Fixed | Coronoid process origin | R / L | Point where the rise of the coronoid process begins on the ramus |
| Fixed | Inferior origin of masseteric crest | R / L | Point where the rise of the masseteric crest begins on the ramus |
| Fixed | Gnathion | Mid | Most inferior and posterior point of the symphyseal groove |
| Fixed | Superior origin of masseteric crest | R / L | Point where the rise of the masseteric crest ends |
| Fixed | Coronoid process head | R / L | Most caudal and superior point on the coronoid process head |
| Fixed | Condylar process head (distal) | R / L | Point on the condylar process head most distal to the centre of the mandible |
| Fixed | Condylar process head (proximal) | R / L | Point on the condylar process head most proximal to the centre of the mandible |
| Fixed | Angular process | R / L | Superior point on the angular process |
| Fixed | Mandibular foramen | R / L | Point inferior to the mandibular foramen |
| Curve | Mandibular body | R / L | Along the inferior curvature of the mandibular body  **[Gnathion - angular process]** |
| Curve | Masseteric crest | R / L | Along the rim of the masseteric crest  [Inferior origin of masseteric crest - superior origin of masseteric crest] |
| Curve | Coronoid process | R / L | Along the height of the coronoid process  [**Origin of coronoid process** to **coronoid process head**] |
| Curve | Mandibular notch | R / L | Along the curve extending from the coronoid to condylar process  **[Coronoid process head - condylar process head (distal)]** |
| Curve | Condylar-angular | R / L | Along the curve extending from the condylar to the angular process  **[Condylar process (proximal) - angular process]** |
| Curve | Condylar process | R / L | Along the midline of the superior surface of the condylar process head  [**Condylar process (proximal)** to **condylar process (distal)**] |
| Curve | Symphyseal groove | Mid | Along the inferior midline of the symphysis [**Gnathion** to **infradentale**] |
| Curve | Superior midline | Mid | Along the superior midline of the symphysis [**Gnathion** to **origin of incisors (superior)**] |
| Patch | Corpus | R / L | Covering the outer surface of the corpus and extending behind the masseteric fossa to the angular process |
| Patch | Masseteric fossa | R / L | Covering the superior/inner surface of the masseteric fossa |
| Patch | Pterygoid fossa | R / L | Covering the fossa enclosed by the inflected mandibular angle in wombats |
