# Supplementary material for: Individual variation of the masticatory system dominates 3D skull shape in the herbivory-adapted marsupial wombats
Source: Front Zool. 2019 Nov 1;16:41. doi: 10.1186/s12983-019-0338-5 (PMC6824091; doi:10.1186/s12983-019-0338-5)
Supplement: Supplementary file 6 — Additional file 6. A detailed description of the landmark variation test we used, corresponding to the text of a vignette for the landvR package. A generalized description, tutorial, and worked examples can also be found on https://github.com/TGuillerme/landvR. [file 12983_2019_338_MOESM6_ESM.docx]

**Additional File 9: Landmark variation details**

The below is a detailed description of the landmark variation test we used, and corresponds to the text of a vignette for the landvR package. A generalized description, tutorial, and worked examples can also be found on <https://github.com/TGuillerme/landvR>.

1. Measuring landmark variation in the Procrustes space (Fig. 1.2)

To measure the magnitude of landmark variation between species, we calculated the Euclidean distances between pairs of corresponding landmarks between the mean Procrustes coordinate positions for the two genera (*Vombatus* and *Lasiorhinus*) and for the two *Lasiorhinus* species. To measure the landmark position variation within groups (i.e. within each species) we compared the distances between pairs of homologous landmarks between the two most distant specimens in Procrustes space (specimens “X” and “Y”), using the following operations:

1. Calculate the distances for all $n$ landmarks between the mean Procrustes coordinates and the coordinates of each specimen.
2. Rank each set of $n$ distances and measure the area under the resulting curve (i.e. the cumulative landmark variation).
3. Select the specimen with the greatest cumulative landmark variation. This is now specimen “X” with the most different shape compared to the mean.
4. Calculate the distances for all $n$ landmarks between specimen “X” and the remaining specimens.
5. Repeat step B and C. The resulting specimen “Y” has the greatest cumulative landmark variation from specimen “X”.

To avoid extreme results due to possible shape outliers, we also ran this algorithm based on the 95% confidence interval (CI) by removing the specimens with the cumulative landmark variation greater than the 97.5^th^ quantile of the distribution.

Note that our approach of comparing the area under the curve of the ranked landmark displacements (i.e. cumulative landmark displacement) is not the same as the assessment of Procrustes differences between specimens. Specifically, using the the cumulative landmark displacement allowed us a finer – grained assessment of shape variation in different regions of the crania and mandibles. For a detailed juxtaposition of the two approaches, see <https://github.com/TGuillerme/landvR/blob/master/inst/vignettes/Procrustes_dist_vs_landmark_integral.Rmd>

1. Testing hypothesis using permutation (Fig. 1.3)

To test whether the selected partitions A) had a significant magnitude of change and B) were from a statistically different configuration of landmarks, we performed random permutation tests between each partition and the entire distribution of landmarks. We did this for the species and genera means and the two most different specimens within each species at 100% and 95% CI.

1. The displacement difference in landmark displacement

The first statistic, termed “displacement difference”, compared the integral of landmark variation distributions of a chosen partition with multiple random partitions (drawn randomly from the entire skull). It describes the difference between the area under the curve from the two distributions of landmark displacements (the one of the focal partition minus the one of the random partition). Displacement difference was calculated:

$Displacement difference=\int_{0}^{n-1} (f_{x}-f_{y})d(x,y)$ (eq. 1)

Where $n$ is minimum number of comparable landmarks and $f_{x}$ and $f_{y}$ are ranked functions (i.e. $f_{0}\geq f_{1}\geq f_{2}...$) for the landmarks in the partition and all the landmarks respectively. If one of the functions $f_{x}$ or $f_{y}$ have $m$ elements (with $m>n$) $f_{z}^{*}$, a rarefied estimate of the function with $m$ elements is used instead.

$\int_{0}^{n-1} f_{z}^{*}d(z)=\frac{\sum_{1}^{p} \int f_{zi}^{*}}{p}$ (eq. 2)

Where $p$ is the number of rarefaction replicates. $p$ is chosen based on the Silverman’s rule of thumb for choosing the bandwidth of a Gaussian kernel density estimator multiplied by 1000 with a result forced to be 100 $\leq p\leq$ 1000 [41].

$p=\left( \frac{0.9\times\sigma_{m}}{1.34n} \right)^{-1/5}$ (eq. 3)

With $\sigma_{m}$ being the minimum of the standard deviation and the interquartile range of the distribution. If the displacement difference is positive, the landmark’s variation in the partition is greater than the overall landmark’s variation, if the difference is negative, the variation is smaller. Magnitudes are only comparable in absolute terms if comparisons come from the same Procrustes superimposition.

1. Probability of landmark displacement overlap

The second statistic, the Bhattacharyya Coefficient, calculates the probability of overlap of two distributions [42, 43]. This is a proxy of the probability of two distributions being the same, for example, whether the landmark displacement in the remainder partition of the cranium are from the same distribution as the landmark displacement from the entire skull. The coefficient is calculated as the sum of the square root of the relative counts shared in $n$ bins between two distributions.

$Bhattacharyya Coefficient=\sum_{i=1}^{n} \sqrt{\sum a_{i}\times\sum b_{i}}$ (eq. 4)

Where $a_{i}$ and $b_{i}$ are the number of counts in bin $i$ for the distributions $a$ and $b$ respectively divided by the total number of counts in the distribution $a$ and $b$ respectively. $n$ was determined using the Silverman’s rule of thumb (see equation 3).

1. Permutation tests for both statistics

We used our two statistics (i.e. displacement difference and Bhattacharyya coefficient) in a random permutation test [44, 45] with $H_{0}$ that the variation in landmark partition is not different than the variation of landmarks in the whole distribution of landmarks in the cranium or mandible. This test aims to calculate whether a potential difference between the observed statistics is different from the same statistic drawn randomly from the same distribution (of landmark displacement, in our case). We generated 1000 statistics by randomly sampling the same number of landmarks as in the partition in the whole distributions and compared them again to the full distribution. This resulted in 1000 null comparisons (i.e. assuming the null hypothesis that the statistic in the partition is the same as the statistic in the total distribution). In other words, we performed the whole procedure (comparing the m subset of landmarks from a partition to the rest of skull) but this time by randomly drawing the m landmarks from the whole skull (rather than just from the partition). We then calculated the *p* value based on:

$p=\frac{\sum_{1}^{B} \left( random_{i}>=observed \right)+1}{B+1}$ (eq. 5)

Where $B$ is the number of random draws (1000 bootstrap pseudo-replicates), $random_{i}$ is the $i^{th}$ statistic from the comparison of the $i^{th}$ randomly drawn partition and the distribution and $observed$ is the observed statistic between the partition and the distribution. Because of the number of tested hypotheses, we lowered our threshold for rejecting $H_{0}$ to 0.001 instead of 0.05.

1. Rarefaction

Because our partitions had different numbers of landmarks (47 “tip of snout” landmarks, 294 “zygomatic arch”, 485 “rest” for the cranium and 47 for “symphysis”, 294 for the “muscle attachment sites”, 293 for the “rest” of the mandible), we rarefied the number of landmarks in the cranium and mandible partitions to 47 in both cases. Thus, for each rarefaction step, we sampled 47 landmark differences per partition *i* following the same procedure as outlined in the permutation test above.
