# Supplementary material for: Individual variation of the masticatory system dominates 3D skull shape in the herbivory-adapted marsupial wombats
Source: Front Zool. 2019 Nov 1;16:41. doi: 10.1186/s12983-019-0338-5 (PMC6824091; doi:10.1186/s12983-019-0338-5)
Supplement: Supplementary file 7 — Additional file 7. Landmarking partitions for testing landmark magnitudes, plotted on the Procrustes mean landmark configuration and cranium/mandible warped to the mean shape. In the cranium (left), blue is the zygomatic arch region with temporal and masseter muscle attachment areas; green is the anterior cranium (“rostrum”), and orange is the remaining landmarks on the cranium. In the mandible, green are the masticatory muscle insertion sites; blue is the anterior symphyseal area; and orange is the remainder of the landmarks on the mandible. Not to scale. [file 12983_2019_338_MOESM7_ESM.pdf]

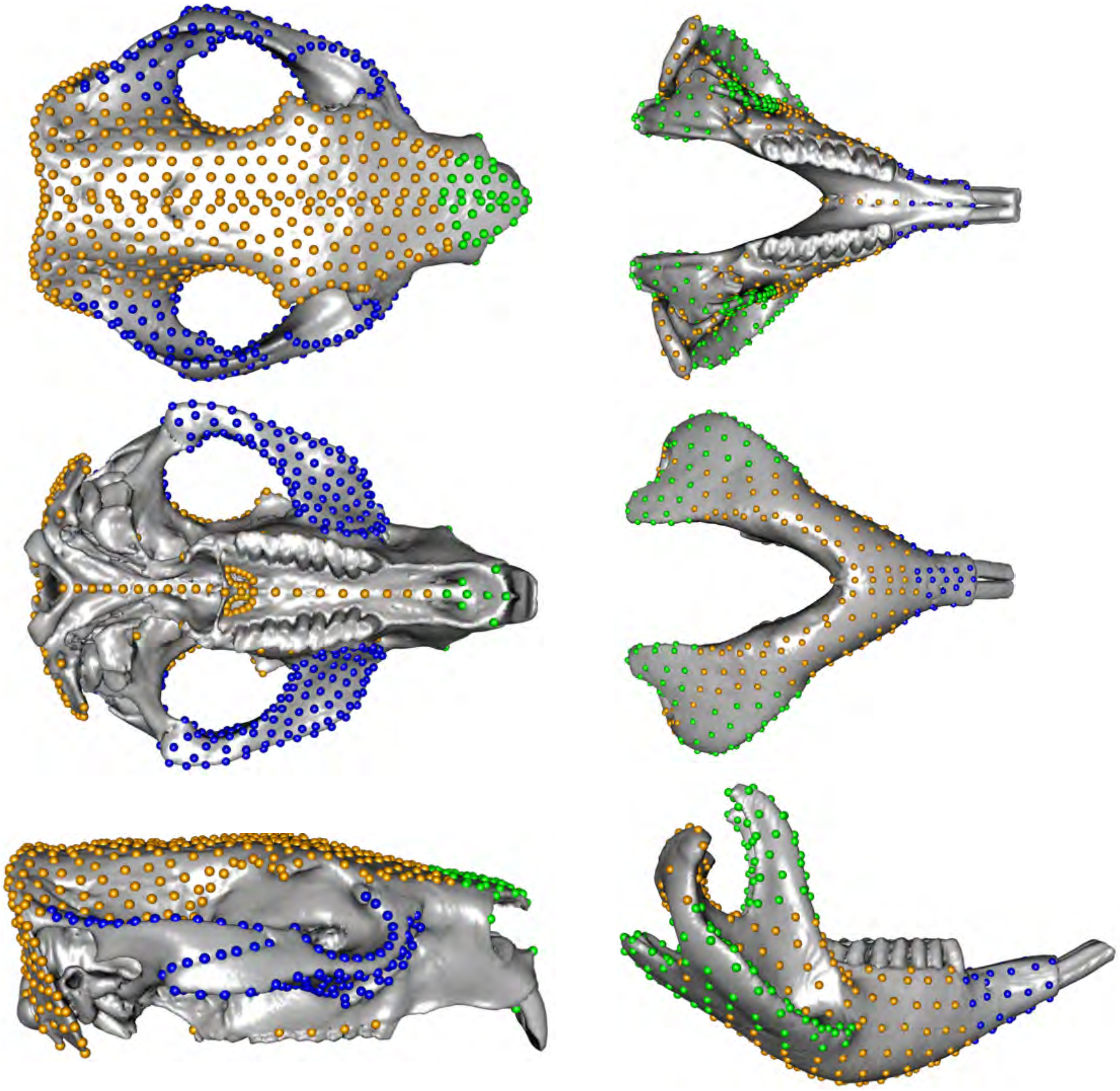

Additional File 10: Landmarking partitions for testing landmark magnitudes, plotted on the procrustes mean landmark configuration and cranium/mandible warped to the mean shape. In the cranium (left), blue is the zygomatic arch region with temporal and masseter muscle attachment areas; green is the anterior cranium (“rostrum”), and orange is the remainder of the skull. In the mandible, green are the masticatory muscle insertion sites; blue is the anterior symphyseal area; and orange is the remainder of the mandible. Not to scale.
